# Identification of Shell Colour Pigments in Marine Snails *Clanculus pharaonius* and *C*. *margaritarius* (Trochoidea; Gastropoda)

**DOI:** 10.1371/journal.pone.0156664

**Published:** 2016-07-01

**Authors:** S. T. Williams, S. Ito, K. Wakamatsu, T. Goral, N. P. Edwards, R. A. Wogelius, T. Henkel, L. F. C. de Oliveira, L. F. Maia, S. Strekopytov, T. Jeffries, D. I. Speiser, J. T. Marsden

**Affiliations:** 1 Natural History Museum, Department of Life Sciences, London, United Kingdom; 2 Department of Chemistry, Fujita Health University School of Health Sciences, 1–98 Dengakugakubo, Kutsukake-cho, Toyoake, Aichi, Japan; 3 Natural History Museum, Imaging and Analysis Centre, London, United Kingdom; 4 School of Earth, Atmospheric, and Environmental Sciences, University of Manchester, Manchester, United Kingdom; 5 NEEM Núcleo de Espectroscopia e Estrutura Molecular, Departamento de Química, Instituto de Ciências Exatas, Universidade Federal de Juiz de Fora, Juiz de Fora, MG, Brazil; 6 Department of Biological Sciences, University of South Carolina, Columbia, South Carolina, United States of America; 7 Viapath, Reference Biochemistry Laboratories, King's College Hospital, London, United Kingdom; University of California, UNITED STATES

## Abstract

Colour and pattern are key traits with important roles in camouflage, warning and attraction. Ideally, in order to begin to understand the evolution and ecology of colour in nature, it is important to identify and, where possible, fully characterise pigments using biochemical methods. The phylum Mollusca includes some of the most beautiful exemplars of biological pigmentation, with the vivid colours of sea shells particularly prized by collectors and scientists alike. Biochemical studies of molluscan shell colour were fairly common in the last century, but few of these studies have been confirmed using modern methods and very few shell pigments have been fully characterised. Here, we use modern chemical and multi-modal spectroscopic techniques to identify two porphyrin pigments and eumelanin in the shell of marine snails *Clanculus pharaonius* and *C margaritarius*. The same porphyrins were also identified in coloured foot tissue of both species. We use high performance liquid chromatography (HPLC) to show definitively that these porphyrins are uroporphyrin I and uroporphyrin III. Evidence from confocal microscopy analyses shows that the distribution of porphyrin pigments corresponds to the striking pink-red of *C*. *pharaonius* shells, as well as pink-red dots and lines on the early whorls of *C*. *margaritarius* and yellow-brown colour of later whorls. Additional HPLC results suggest that eumelanin is likely responsible for black spots. We refer to the two differently coloured porphyrin pigments as trochopuniceus (pink-red) and trochoxouthos (yellow-brown) in order to distinguish between them. Trochopuniceus and trochoxouthos were not found in the shell of a third species of the same superfamily, *Calliostoma zizyphinum*, despite its superficially similar colouration, suggesting that this species has different shell pigments. These findings have important implications for the study of colour and pattern in molluscs specifically, but in other taxa more generally, since this study shows that homology of visible colour cannot be assumed without identification of pigments.

## Introduction

Colour plays an important role in the survival of many species, specifically in mate attraction, signalling, camouflage, thermoregulation, immunity and strengthening [1–4]. Colour can also offer a visible link between an external phenotype and its corresponding genotype, providing a model for the exploration of fundamental evolutionary processes [2]. In order to study how colour and pattern have evolved in any organism it is imperative to know which pigments are responsible for colouration and how these pigments originate [4–6]. There are several classes of biological pigments, and each can give rise to a range of colours that overlaps with those produced by other pigment classes [4, 7]. As such, it is not possible to identify the pigment classes present in any organism purely by its visible colour, making it essential to identify pigments biochemically to avoid mistaking homoplasy for homology. Once a pigment is known, molecular studies can be more targeted in identifying genes associated with its pigment [4].

Molluscs are often renowned for their highly colourful shells, and shell colour and pattern have proved to be highly tractable markers for use in studies on natural selection, in part because shells and shell fragments can be easily collected [4] (e.g. [8]). Recently, there has been growing interest in the molecular origins of shell colour (e.g. [9–12]). The biochemical nature of shell pigments was investigated by a number of researchers towards the end of the 1800s, reaching a peak in terms of number of studies in the mid-1900s (e.g. [5, 13–24]). Older studies identified two main classes of shell pigments: melanins and tetrapyrroles (which include porphyrins and linear tetrapyrroles known as bile pigments or bilins) along with a number of other pigments that could not be fully characterised, some of which were complexed to proteins. More recent chemical studies are scarce, but have confirmed the presence of porphyrins, bilins and incompletely characterised pigments identified only as polyenes (organic compounds containing multiple double bonds) (e.g. [24–26]).

However, the extraction and identification of pigments is extremely difficult. For example, in one recent study, more than 30 kg of shells of *Haliotis discus hannai* were required to obtain only 2.7 mg of purified pigment [24]. Such studies are likely only possible for species that are very large or the target of aquaculture (as in the case of *Haliotis*). In part because of the difficulties associated with chemical identification of shell pigments, many recent studies of shell pigmentation have focused on using Resonance Raman Microspectrometry to examine whole shells. These studies have also found polyenes or polyenes bound to other molecules in shells, which are usually described as carotenoids, and occasionally as psittacofulvins (e.g. [7, 27–30]).

Although little is known about the evolution of pigments in molluscs, it seems likely that pigment types are distributed in a phylogenetically relevant manner ([4, 15] and references therein). For instance, tetrapyrrole pigments such as porphyrins and bilins occur most commonly in vetigastropods and have not been found in the shells of freshwater gastropods (other than some neritids) or land snails [20]. Conversely, polyenes (mostly thought to be carotenoids) have been identified by Raman Microspectrometry primarily in shells of caenogastropods, hetereobranch gastropods and unionid bivalves [28, 29, 31–34].

**Fig 1:**
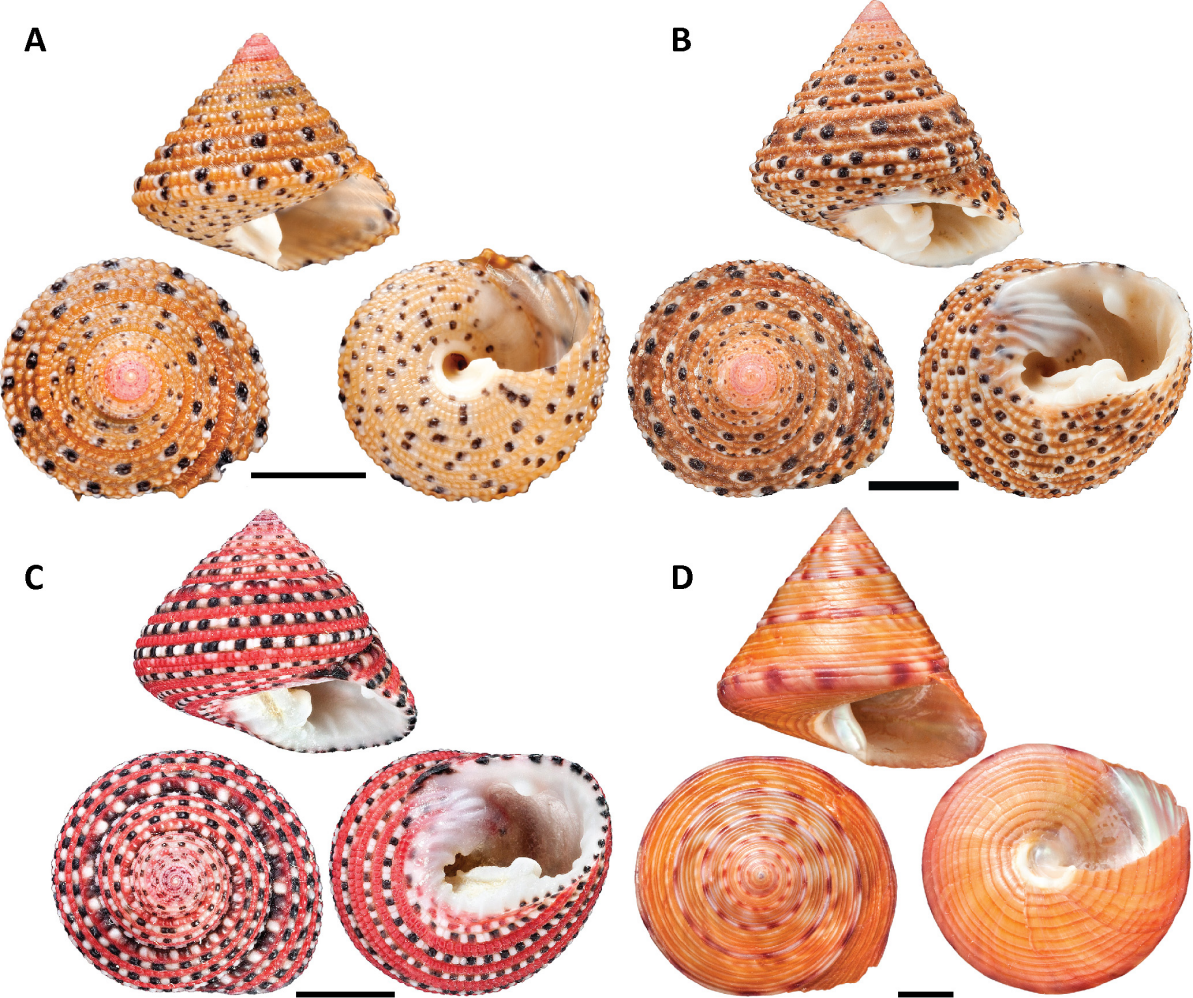
Photos of exemplar specimens. (A) *Clanculus margaritarius* A. Shell from Kitahama, Japan. Note that the specimen is sub adult. Specimen # 3. (B) *Clanculus margaritarius* B. Shell of an unlocalised specimen, NHMUK collection. Specimen # 10. (C) *Clanculus pharaonius*. Rose Reef, Thuwal, Saudi Arabia. Specimen # 4. (D) *Calliostoma zizyphinum*. Shetland Islands, United Kingdom. Specimen # 1. Scale bars = 5 mm, except in (D), where scale bar = 1 cm.

In this study we use a variety of chemical and multi-modal spectroscopic techniques to identify and characterise as fully as possible shell pigments in two nominal species of the vetigastropod genus, *Clanculus* (Trochoidea; Trochidae): *Clanculus margaritarius* (Philippi, 1846) (Fig 1A and 1B) and *C*. *pharaonius* (Linnaeus, 1758) (Fig 1C). *Clanculus pharaonius* shells are a vivid pink-red with black and white spots and *C*. *margaritarius* shells have pink-red spots and lines on early whorls, but yellow-brown on later whorls, again with black and white spots as seen in *C*. *pharaonius*. We compare these results with those obtained for another vetigastropod from the same superfamily *Calliostoma zizyphinum* (Linnaeus, 1758) (Trochoidea: Calliostomatidae), (Fig 1D). *Calliostoma zizyphinum* shells have both pink-red and yellow-brown markings, similar to the colours seen in the *Clanculus* species. Identification of the pigments in these three species provides the opportunity to determine whether their superficially similar colouration arises from homologous pigments as well as providing a foundation for molecular studies targeting genes associated with pigment production in this superfamily.

## Materials and Methods

### Samples

Specimens used in this study included both live-collected specimens of *Clanculus margaritarius* A from Japan and dry, unlocalised shells of *C*. *margaritarius* B from NHMUK collections (Table A in S1 File). The latter differed slightly from the former by small differences in the colour pattern and shell sculpture, notably on early whorls, and the two forms may represent sibling species. *Clanculus pharaonius* were all collected from Saudi Arabia. *Calliostoma zizyphinum* specimens came from the United Kingdom (See Table A in S1 File for more information). Specimens from the UK and Japan were donated to the NHMUK and no specific permits were required to collect these samples. Samples from Saudi Arabia were collected in association with the King Abdullah University of Science and Technology (KAUST) and access to local coral reefs was granted by the Saudi Arabian Coast Guard. Given that the research involved invertebrates, no approval was required by the KAUST Institutional Animal Care and Use Committee. The species in this study are neither endangered nor protected. Voucher specimens of each species have been deposited at the Natural History Museum (London) (see Table A in S1 File).

Methods used to identify pigments were undertaken first with *C*. *margaritarius* and, if informative, repeated for *C*. *pharaonius*. Methods that were useful for identifying the pink-red and yellow-brown coloured regions were repeated for *Ca*. *zizyphinum*. All shells were cleaned in an ultrasonicator with mild detergent prior to analyses.

### High performance liquid chromatography (HPLC) analyses

#### Fluorescent pigments—uroporphyrin I and III

Previous studies have shown that *Clanculus pharaonius* shells fluoresce red under UV light and have identified the pigment uroporphyrin I in shells using chromatography [35, 36]. In this study we exposed dry shells of *C*. *margaritarius*, *C*. *pharaonius* and *Ca*. *zizyphinum* to short- and long-wave UV light (254 and 366 nm) to identify any fluorescence. Of our target pigments, only porphyrins and bile pigments fluoresce under UV light [37, 38]; however, if these pigments are complexed to a protein moiety, fluorescence is quenched [39].

Whole shells from two specimens of *C*. *margaritarius* and one specimen of *C*. *pharaonius*, and a 1cm^2^ piece of *Ca*. *zizyphinum* shell with both pink-red and yellow-brown colours were analysed by HPLC using a Dionex UltiMate 3000 system with a Dionex RF2000 fluorescence detector to identify the fluorescent compounds. Shells were broken and decalcified separately with concentrated hydrochloric acid (37%) for 24 hours at room temperature in the dark and diluted at least one in five with deionised water prior to HPLC analysis. Foot tissue from both *Clanculus* species was treated in the same manner. HPLC separation was carried out on a Poroshell C18 column (2.7μm, 4.6 x 50mm; Agilent Technologies, UK). A 10 μL aliquot of the solution was injected onto the column at a flow rate of 1.2ml/min. Porphyrins were detected using fluorescence with an excitation wavelength 404 nm and emission wavelength 618 nm, which is characteristic of porphyrins.

To measure retention times, gradient elution of a standard mixture containing the porphyrins uroporphyrin I, uroporphyrin III, hepta-, hexa- and pentacarboxylate porphyrins, coproporphyrin I, coproporphyrin III, deuteroporphyrin and mesoporphyrin was carried out with Solvent A: 1 M ammonium acetate buffer pH 5.16: acetonitrile (90:10) and Solvent B: methanol: acetonitrile (90:10). The standard mix was obtained by combining a porphyrin marker kit containing uroporphyrin I, hepta-, hexa- and pentacarboxylate porphyrin I, coproporphyrin I and mesoporphyrin IX from Frontier Scientific Ltd, UK with uroporphyrin III, coproporphyrin III, deuteroporphyrin IX and protoporphyrin IX from Sigma-Aldrich Ltd, UK. Sample porphyrins were separated with a 15 min linear gradient elution from 100% Solvent A to 10% Solvent A (90% Solvent B) followed by isocratic elution at 90% Solvent B for a further 3 mins. The gradient then reverted to 100% Solvent A. An additional check for porphyrin peak identity was undertaken by running two commercially available quality control samples; ClinChek Levels I and II (Recipe Chemicals and Instruments, Germany). These were included at the beginning and the end of all assays and contained known amounts of uroporphyrin I, hepta-, hexa- and penta-carboxylate porphyrin, coproporphyrin I and coproporphyrin III.

Pyrromethanes can form uroporphyrin I under acidic conditions [40], so we repeated HPLC analyses with a modified protocol to confirm our results. One *C*. *margaritarius* shell (specimen # 12) was partially dissolved in 0.5 M Na-EDTA pH 8.0 at 65°C. After 1.5 h the top layer of the shell had dissolved and holes were starting to form in the shell. Some orange-pink pigment remained in the early whorls, although the early whorls themselves were dissolving. The pale brown suspension containing the pigment was passed through a Bond Elut C18 column (Analytichem International, part 607203). A brown layer that could be seen on the filter did not elute with water, but eluted with methanol. This sample was also run on the HPLC under the same conditions as above.

#### Non-fluorescent pigments–melanin

We hypothesised that black-coloured spots on the shells of both *Clanculus* species might be due to the common pigment melanin. Four shells of *C*. *margaritarius*, including one shell that had the top pigmented layer removed, and one shell of *C*. *pharaonius* were decalcified by gently mixing for 30 min in 20 volumes (v/w) of 1 M HCl. Aliquots of 1.5 mL of the suspensions obtained were placed in 10 mL screw-capped conical test tube and evaporated to dryness in a vacuum desiccator. The residues were subjected to alkaline H_2_O_2_ oxidation to convert melanin to pyrrole-2,3,5-tricarboxylic acid (PTCA), a specific eumelanin marker, as described in [41]. In brief, 100 μL water, 375 μL M K_2_CO_3_ and 25 μL 30% H_2_O_2_ were added to the test tubes containing the seashell preparation. The tubes were mixed vigorously at 25° ± 1°C for 20 h. The residual H_2_O_2_ was decomposed by adding 50 μL 10% Na_2_SO_3_ and the mixture was then acidified with 140 μL 6 M HCl. Each reaction mixture was briefly centrifuged, and an 80 μL aliquot of each supernatant was directly injected into the HPLC system as described in [41]. The presence of 4-amino-3-hydroxyphenylalanine (4-AHP), a specific pheomelanin marker, was determined after reductive hydrolysis with hydroiodic acid as described in [42].

### Confocal microscopy

We examined fluorescence of the shells of eight specimens of *C*. *margaritarius* including the specimen with the top layer of pigmented shell removed (specimen # 12), two specimens of *C*. *pharaonius* and one specimen of *Ca*. *zizyphinum* using a confocal microscope to determine whether fluorescence was congruent with visible pigment. Confocal images were acquired with a Nikon A1-Si laser-scanning confocal microscope using a 10x objective (NA 0.3). Images were recorded with pixel dimensions of 1.24 μm. To investigate the presence and distribution of uroporphyrin I and uroporphyrin III, we used a spectral detector to acquire a complete fluorescence emission spectrum. Autofluorescence of the shell was excited with a 405 nm line of 100 mW cube laser, a 488 nm line of 50 mW sapphire laser, a 561 nm line of 50 mW sapphire laser and a 640 nm line of 40 mW cube laser (all lasers by Coherent Inc., USA). The fluorescence signal was collected with a 32 channel spectral detector at 10 nm resolution on a diffraction grating covering a range of wavelengths between 410 nm and 730 nm. The shell surface was visualised using a 34.5 μm (1.2 airy units) confocal pinhole and a number (typically between 60 and 130) of z-stacks were acquired, each with optical thickness of 7.5 μm. Emission spectra were acquired with the same microscope setup for uroporphyrin I dihydrochloride and uroporphyrin III dihydrochloride standards (Frontier Science). The fluorescence signal collected from raw images was then spectrally unmixed using these reference data and Nikon NIS-Elements software (http://www.nis-elements.com/).

### UV-visible spectrophotometry

Pigments were extracted from one shell fragment of each species: *C*. *margaritarius* and *C*. *pharaonius* by ca. 5 ml of 0.5 M Na-EDTA (pH 8) for 1.5 h at 65°C. The extracts were filtered through 0.2 μm polypropylene syringe filters and their spectra (300–900 nm with a resolution of 0.5 nm) were recorded using a Shimadzu UV-1800 UV-visible spectrophotometer in 1 cm polystyrene cells against Na-EDTA solution as a blank. After the initial exposure to light for ca. 3 h, the solutions were kept in darkness for 13 days and their spectra were re-recorded to check the stability of the extracted pigments in the near-neutral Na-EDTA solution.

### Mass spectrometry

We attempted to further identify porphyrin pigments from three samples of *C*. *margaritarius* shell using positive ion electrospray mass spectrometry. Shell pieces containing pigment were treated with either 6 M HCl or 0.5 M EDTA at room temperature for 2 h. The supernatants were passed onto a prewashed Bond Elut C18 column where a band of pigment was observed at the top of the column. The columns were washed with water and then UV-absorbing material was then eluted with methanol followed by methanolic ammonium hydroxide (90:10 v/v). Solvents were removed under a stream of nitrogen, and the samples re-suspended in 50% methanol:water for analysis by positive ion electrospray mass spectrometry on a Micromass Quattro II instrument; however, there was insufficient material available to obtain structural information.

### Raman spectroscopy

The yellow-brown surface and black spots of two *C*. *margaritarius* shells were examined using microscopic analyses with Raman spectroscopy. Multiple areas were analysed on each shell. The shell was illuminated by light with a wavelength of 532 nm using a Senterra Bruker instrument. Laser output at the source was 1–10 mW.

### Trace metals associated with pigmentation

Pigments like porphyrins and melanin are often associated with metal ions so we used a suite of methods to determine whether any metals were associated with the shell pigment patterns observed in *C*. *margaritarius*.

#### Energy dispersive spectrophotometry (EDS)

We used EDS to test for the presence of trace metal ions on the shell surface. Analyses at the Natural History Museum were carried out using a LEO1455VP scanning electron microscope equipped with a X-Max 80 mm^2^ EDS detector (Oxford Instruments Ltd.) using the following microscope setup: 20 kV, I probe = 447 pA, chamber pressure 20 Pa, working distance 15 mm. The acquisition time was set to 120 s and ~25% dead time. These data were compared with analyses run at the University of Manchester on a FEI Quanta 650 FEG SEM equipped with a multi-channel Bruker 5060F XFlash QUAD EDS system with an acquisition time of 180 s, working distance 8.8 mm, chamber pressure 67 Pa and an accelerating voltage of 25 kV.

#### Microfocus synchrotron analysis

Synchrotron methods have been successfully used for the analysis of biological soft tissues for over 30 years, see [43] for a review of microfocus applications. Maps and sulphur X-ray Absorption Near Edge Structure (XANES) spectra were obtained at Diamond Light Source microfocus Beamline I18 using Kirkpatrick-Baez mirrors to produce a spot size of approximately 5 μm, a double crystal Si (111) monochromator to scan incident beam energy, and a 4-element Vortex silicon drift detector positioned at 90 degrees to the incident beam to detect the X-ray fluorescence (XRF) signal. Flux was estimated to be approximately 3 x 10^10^ photons s^-1^. Scan speed was either 0.10 or 0.21 s pixel^-1^. With this system, a full EDS spectrum is recorded for every pixel of the XRF map. EDS fluorescence point spectra were also obtained by counting for 30 seconds at a specific location. Each spectrum was fit using the PyMCA freeware [44] from fundamental parameters of the experiment using a Durango apatite mineral standard with known element concentrations for calibration. Different areas were analysed in triplicate and the results averaged. Maps of heavy elements (K-Sr) were collected under ambient conditions. Maps of light elements (Al-Cl) and sulfur X-ray Absorption X-ray Absorption Near Edge Structure (XANES) spectra were recorded by placing the sample and detector aperture into a helium-purged bag to minimize absorption of the incident and fluoresced signals. A ZnSO_4_ standard was used to calibrate the energy of the monochromator.

#### Fourier transform infrared (FTIR) spectra

FTIR spectra were collected by attenuated total reflectance Fourier transform infrared spectroscopy (ATR-FTIR) using a Perkin Elmer Spotlight 400 imaging system. Spectra were taken using a wavenumber range of 4000 to 650 cm^-1^, at a resolution of 4 cm^-1^ and using an aperture of 100 μm^2^. Sixteen replicate spectra were obtained. Each was background subtracted and then all were summed to produce an averaged, background subtracted, summary spectrum. Organic peak assignments were made using the Bio-Rad KnowItAll Informatics System 8.2 Multi-Technique database. Uroporphyrin I ethyl ester from bovine porphyric urine (CAS number: 54090-85-6) was purchased from Sigma Aldrich and used as a standard for comparison to the shell.

#### Time-of-Flight secondary ion mass spectrometry (ToF-SIMS)

ToF-SIMS with C_60_ primary ions was used for comprehensive analysis of inorganic and organic material of the shell surface. ToF-SIMS uses a pulsed primary ion beam to sputter material from the surface which is then analysed using a time-of-flight mass analyser giving a full mass spectrum up to mass 2000. The ToF-SIMS instrument, which is now equipped with a C_60_ primary ion source, is described in detail in [45] and a more general overview of ToF-SIMS is given in [46]. C_60_ primary ions are ideally suited for organic analysis because it is possible to overcome the static limit [47] which means that the sample surface can be cleaned from surface contamination without destroying organic material in the sample. C_60_ primary ions are also very suitable for inorganic analyses because of accuracy improvements for quantitative analyses [48].

Using a primary ion beam of 10 pA, the sample was first cleaned with a dc-beam over a 300 μm area to remove any surface contamination from sample handling. The analysis was then performed with a pulsed beam of 10 ns pulse duration and an area of 250 μm with 256 × 256 pixels. Secondary ion images have been extracted from peaks marked in the total mass spectrum and sub-regions have been defined for semi-quantitative analysis. High mass resolution (M/dM > 5000) was achieved by using a delayed extraction method to ensure that molecular interferences could be resolved.

#### Laser ablation inductively coupled plasma mass spectrometry

All the above methods rely on surface analyses, so we also undertook laser ablation inductively coupled plasma mass spectrometry (LA-ICP-MS), which produces depth-resolved data. Laser ablation was used to examine *C*. *margaritarius* and *C*. *pharaonius* specimens.

Qualitative analyses of 25 trace elements were performed by LA-ICP-MS using a New Wave Research NWR193 laser coupled to an Agilent Technologies 7500cs ICP-MS system. Determinations were performed in a He atmosphere. Background gas signals were collected for 35 s followed by 55 s of ablating the samples. The laser was fired at 5Hz, using a beam diameter of 45 μm and fluence of 2.8 J cm2. Data were collected using a peak jumping time resolved acquisition technique. The data for the output between 40 s and 60 s were normalised to the counts for calcium and plotted to determine any variation between the exhibited colours. The method was used to examine multiple samples of each colour (yellow-brown or red, black and white) on three shells of *C*. *margaritarius* and two shells of *C*. *pharaonius*.

## Results

### Fluorescent pigments–uroporphyrin I and III

**Fig 2:**
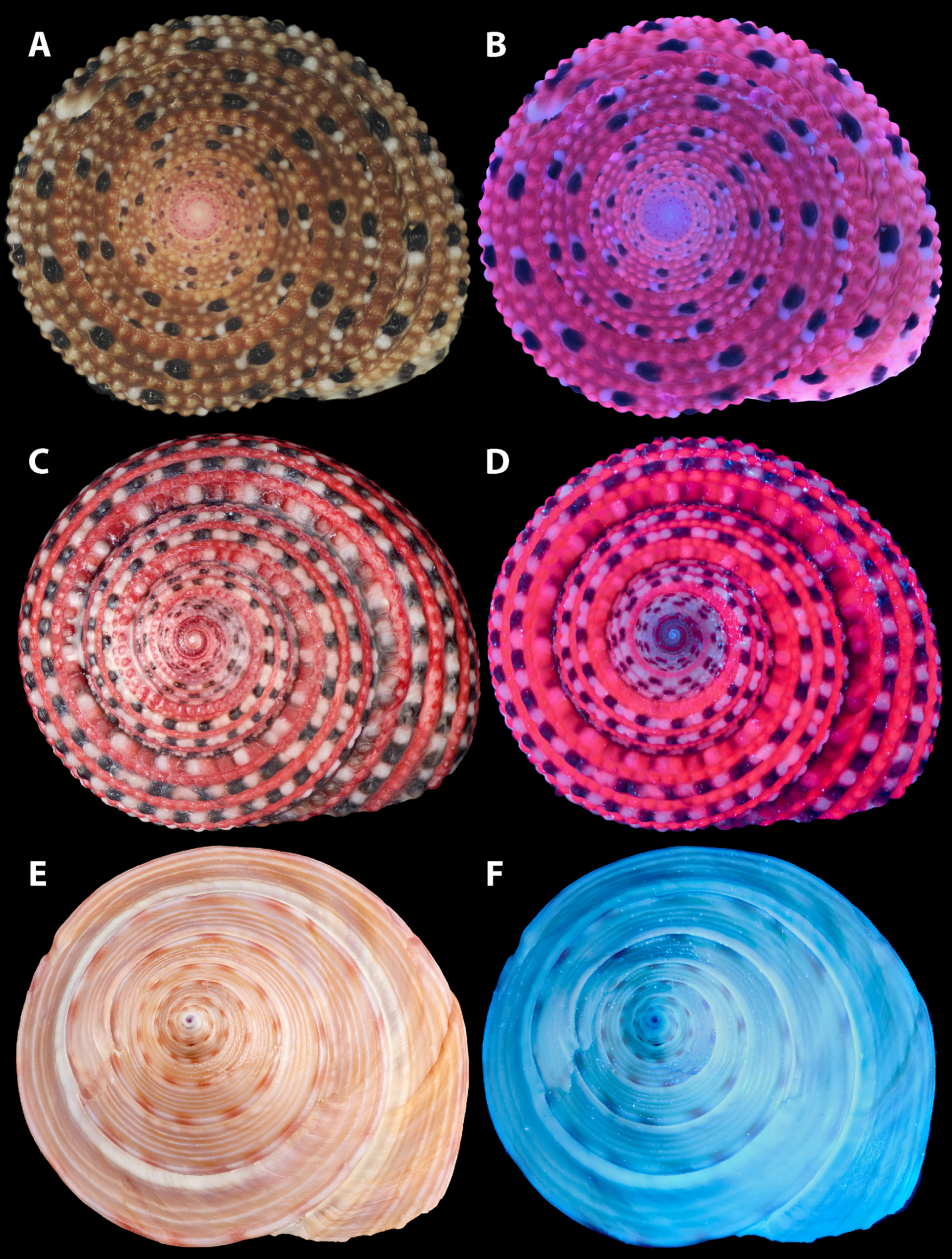
Shells under visible and ultraviolet (UV) light. (A, B) *Clanculus margaritarius* A (specimen #7), NHMUK 20150502. (C, D) *Clanculus pharaonius* (specimen #2). NHMUK 20160357. (E, F) *Calliostoma zizyphinum* (specimen #1). NHMUK 20160315. (A, C, E) Visible light. (B, D, F) UV light. Note that both the pink-red and yellow-brown pigmentation of *Clanculus* shells fluoresce red under UV light. The *Ca. zizyphinum* shell does not fluoresce under UV light.

All *Clanculus* specimens fluoresced red under UV light but no conspicuous fluorescence was observed for *Ca*. *zizyphinum* under UV light (Fig 2). A *C*. *margaritarius* shell that had the top pigmented layer removed through decalcification by Na-EDTA, did not show strong fluorescence, indicating that fluorescence was due to pigments found only in the top layer of shell.

**Fig 3:**
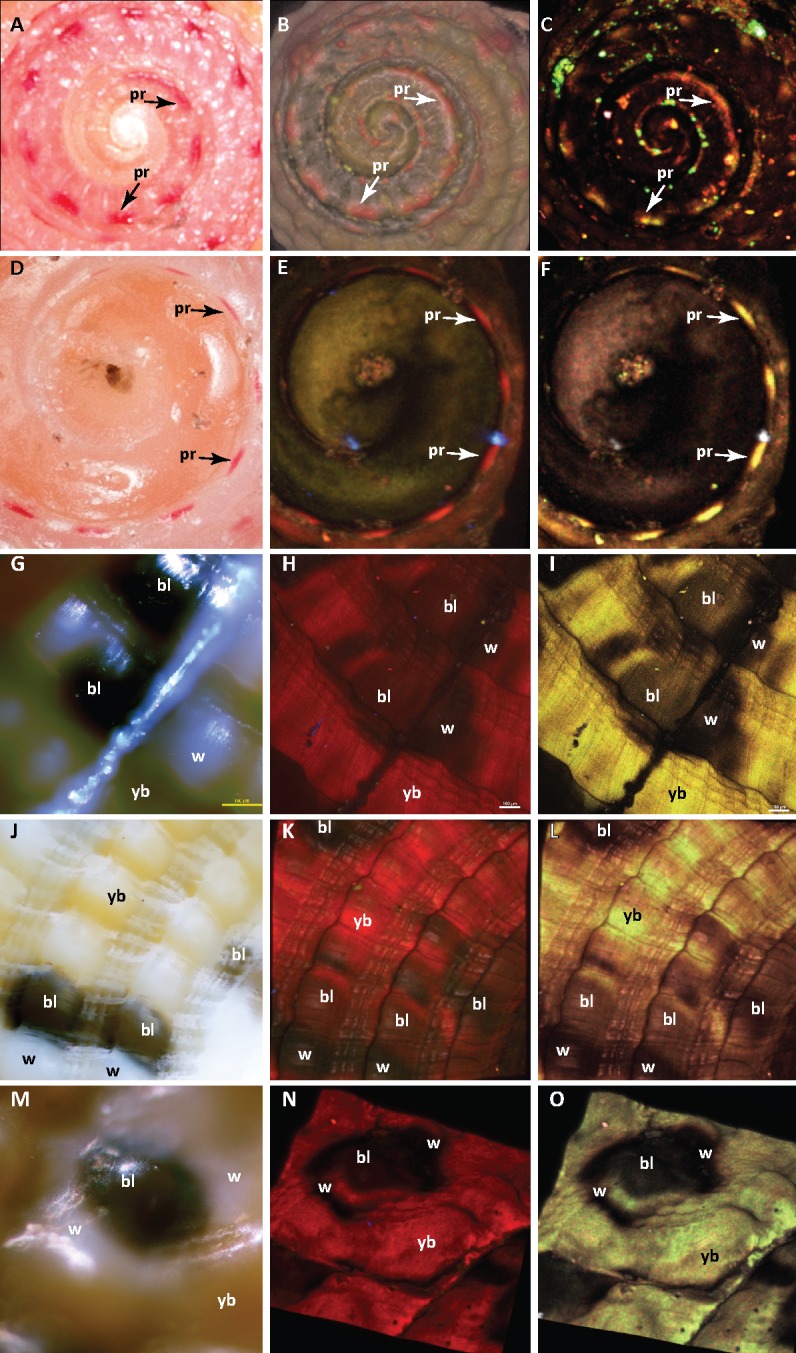
Confocal images showing uroporphyrin distribution in areas of pink-red and yellow-brown pigmentation on *Clanculus margaritarius* shell. Shell images on each line are of a single shell and are in approximately the same orientation and magnification. (A-C) Top of shell, *Clanculus margaritarius* A (specimen #2). (A) Visible pink-red pigmentation. (B) Shaded volume 3D projection of a spectrally mixed image of the same shell showing fluorescent red areas congruent with visible pink-red pigment. (C) Maximum intensity projection of a spectrally unmixed image showing distribution of uroporphyrin I and III. (D-F) *Clanculus margaritarius* B (specimen #10). (D) Visible pink-red pigmentation. (E) Maximum intensity projection of a spectrally mixed image showing fluorescent red areas under UV light congruent with pink-red pigment. (F) Maximum intensity projection of a spectrally unmixed image showing distribution of uroporphyrin I and III. (G-L) Two different areas with yellow-brown, black and white colouration on shell of *C*. *margaritarius* A (specimen #2). (M-O) Yellow brown areas on shell of *Clanculus margaritarius* B (specimen #10). (G, J, M) Visible pigmentation for approximately the same areas as used in analyses. (H, K, N) Maximum intensity projection of spectrally mixed images showing red fluorescent areas under UV light congruent with yellow-brown pigmentation. Panel N is shown in 3D. (I, L, O) Maximum intensity projection of spectrally unmixed images showing distribution of uroporphyrin I and III. Panel O is shown in 3D. Note that in unmixed images green corresponds to uroporphyrin I and red to uroporphyrin III and yellow to areas where both co-occur. Note also that fluorescence is quenched in black spots and nearby white areas. Visible pigmentation is marked on each image: pr–pink-red, bl–black spot, w–nearby white area, yb–yellow-brown. Panels (G-I) include a small strip of blu-tack used to locate coloured areas.

**Fig 4:**
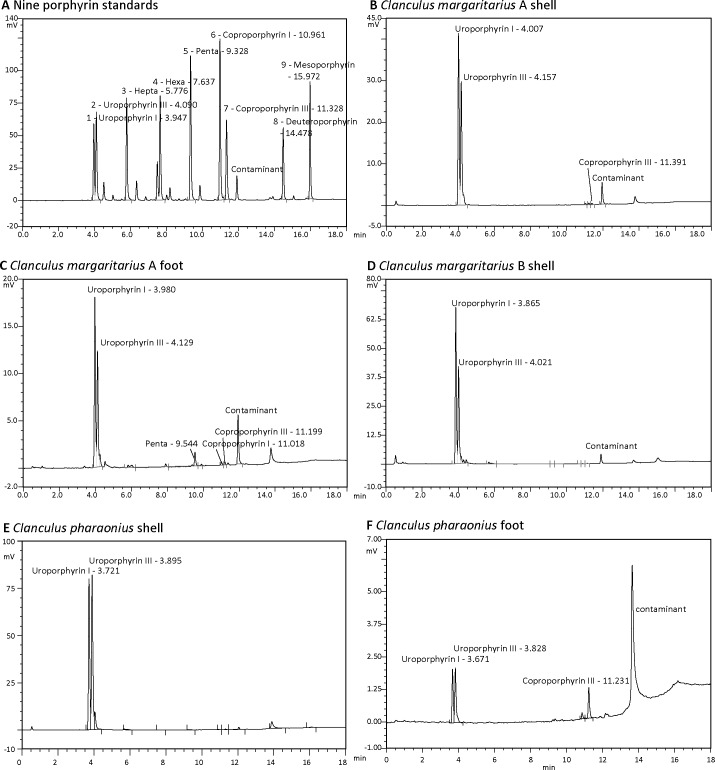
HPLC analyses for porphyrins in *Clanculus*. (A) Chromatogram of nine porphyrin standards. (B, C) *Clanculus margaritarius* A. (B) Chromatogram of shell extract (specimen #3, 1:20 dilution). (C) Chromatogram of extract from coloured foot tissue (preserved in RNALater) (specimen #3). (D) Chromatogram of *C*. *margaritarius* B shell extract (specimen #9, 1:5 dilution). (E) Chromatogram of shell extract of *Clanculus pharaonius* (specimen #2). (F) Chromatogram of extract from foot tissue of *Clanculus pharaonius* (specimen # 1). Note that (B) and (C) were run on the same day as the standards shown in (A); other samples were run on different days.

Red fluorescence under UV light suggested the presence of porphyrins in the shell of *C*. *margaritarius* (Figs 2 and 3) and *C*. *pharaonius* (Fig 2, Figure B in S1 File), which was confirmed by HPLC analyses. Two large peaks in the HPLC traces corresponding to uroporphyrin I and uroporphyrin III were observed, although the peak area ratio differed between the two species (Fig 4). Retention times of the porphyrins from the shell extracts showed very slight drift during HPLC analysis when compared with standards. A recognised explanation for this is that the analysis was run overnight and the ambient temperature of the laboratory fluctuates during the evening. Temperature fluctuation can affect evaporation of the organic phase affecting the retention times of the porphyrins eluting from the column. The use of internal quality controls run on all assays at the beginning and the end of each run ensures that peaks were identified correctly, even in the presence of slight drift.

Trace amounts of coproporphyrin III were also identified via HPLC in *C*. *margaritarius* shell. Results of HPLC analyses of *C*. *margaritarius* and *C*. *pharaonius* foot tissue also identified uroporphyrin I and III and trace amounts of coproporphyrin I and pentacarboxylate porphyrin. Analysis of pigment dissolved from the top layer of a *C*. *margaritarius* shell without the use of concentrated acid (using Na-EDTA instead) showed a similar result, with trace amounts of coproporphyrin III and heptacarboxylate porphyrin (data not shown). No obvious fluorescence was observed for a *Ca*. *zizyphinum* shell viewed under a UV light and only trace amounts of uroporphyrin were identified using HPLC (data not shown).

Examination of shells using a confocal microscope to detect fluorescence associated with uroporphyrins showed that the distribution of uroporphyrin I and III in *Clanculus* shells was congruent with pink-red spots and areas of yellow-brown pigmentation on *C*. *margaritarius* shells and with pink-red areas on *C*. *pharaonius* shells (Fig 3, Figure B in S1 File). There was no fluorescence associated with uroporphyrin I and III in black spots on the shell and there appeared to be none at all in white areas closely associated with the black spots. The cream-coloured, iridescent, nacreous layer of shell underlying visible pigmentation showed very weak red fluorescence suggesting that the uroporphyrins occur in the top, pigmented layer of the shell. There was no fluorescence associated with pigmentation in *Ca*. *zizyphinum* shells.

### UV-visible absorption spectroscopy

Porphyrins display characteristic absorption and fluorescence properties in the visible region. Porphyrins have a wavelength of maximum absorption (λ_max_) around 400 nm, known as the Soret band, and weaker absorption bands, known as Q-bands, between 450 and 700 nm [49]. There are four Q-bands in metal-free porphyrins as their free bases and only two in porphyrins complexed to metal ions [50]. There are four types of porphyrin (*etio*-, *phyllo*, *rhodo*- or *oxorhodo*), which differ in their substituents; different substituents can result in different colours [49, 50]. The type of porphyrin can be determined by the relative heights of Q-bands.

**Fig 5:**
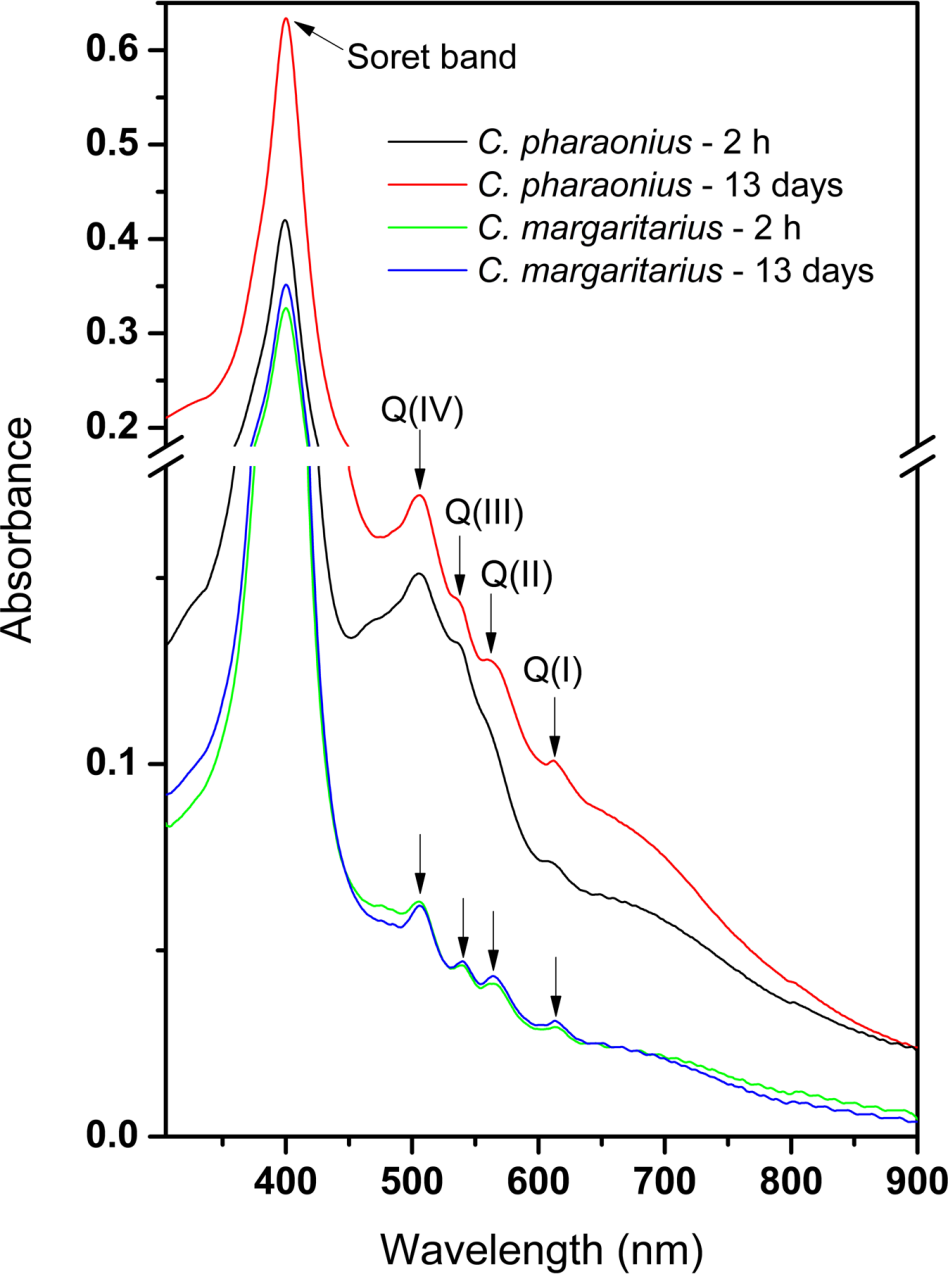
UV-visible absorption spectra of the Na-EDTA aqueous extracts from *C*. *margaritarius* A (specimen #5) and *C*. *pharaonius* (specimen #5) collected at 2 hours and 13 days after extraction. Positions of λ_max_ (Soret band) and Q bands Q(I)–Q(IV) are marked.

The colour of the Na-EDTA extract from the shell of *C*. *margaritarius* was brown and stable over 13 d, whereas fresh pigment extract for *C*. *pharaonius* was red, but turned red-brown over time (Fig 5). Examination of Na-EDTA extracts from the shells of both *Clanculus* species over this time period show features characteristic for porphyrins in their UV-visible absorption spectra: Soret bands at 400 nm and four Q-bands, typical for metal-free porphyrins as their free bases [50]. The spectrum of the *C*. *margaritarius* extract is characterised with the following positions of Q-bands: I– 613 nm, II– 564 nm, III– 539 nm and IV– 506 nm. It is not easy to see whether it is *etio*-type with the order of intensities is IV > III > II > I or *phyllo*-type with IV > II > III > I [50]. The spectrum of the *C*. *pharaonius* extract shows the same positions of Q bands (except that Q(II) is just a tiny shoulder peak); it is an *etio*-type with the order of intensities of IV > III > II > I. These values differ slightly from those found by Tixier [36], which were I– 625 nm, II– 572 nm, III– 532 nm and IV– 500 nm, but the positions of bands depend on solvents and so are not expected to be identical. There is also an additional broad absorption band in the 450–600 nm region and a shoulder at ca. 470 nm. Aging for 13 days in aqueous solution at pH 8 causes both *C*. *margaritarius* and *C*. *pharaonius* extracts to become *phyllo*-type (also the shoulder at 470 nm in the *C*. *pharaonius* extract disappears). Thus the porphyrin type changes over time, which may explain the colour change observed for *C*. *pharaonius* pigment extracts.

### Raman spectroscopy

*In situ* spectra excited by 532 nm light showed a collection of bands at approximately 1556, 1445, 1335, 1085 and 703 cm^-1^ (Figure C in S1 File) for the yellow-brown regions of the shells. Spectra of the black spot showed bands at approximately 1554, 1443, 1345, 1109, 1085 and 703 cm^-1^ (Figure C in S1 File). Raman bands at 1085 and 703 cm^-1^ [n(CO_3_^2-^)] can be positively assigned to the CaCO_3_ polymorph aragonite [27, 51]. The Raman band at 1555 cm^-1^ observed in the *in situ* spectrum of the *C*. *margaritarius* shell can be assigned to the vibrational (C_β_-C_β_) stretching mode assigned to pyrrole systems in porphyrins [29, 52, 53]. However, this band could also be attributed to C = C stretching mode of conjugated polyenes with 7 double bonds [7, 34, 54]. Spectra from the yellow-brown area and from the dark spot showed similar Raman bands, with dark spots showing a slight enhancement of Raman signal (Figure C in S1 File).

### Identification of black pigment–eumelanin

HPLC analyses confirmed the presence of PTCA, a specific eumelanin marker, but did not detect 4-AHP, a specific pheomelanin marker in *Clanculus* shells (Table 1). PTCA was detected, although only at trace levels, in pigmented *Clanculus* shells, but not in a shell that had the top pigmented layer removed through decalcification by Na-EDTA, confirming that pigment occurs only in the top layer of the shell. The specific pheomelanin marker 4-AHP was not detected. HPLC traces for specimens *Clanculus margaritarius* B #8 and #12 are shown to illustrate results (Figure D in S1 File)

**Table 1:** Concentration of melanin markers in *Clanculus* shells determined by HPLC.

Specimen	PTCA (ng/g)	4-AHP (ng/g)
*Clanculus margaritarius* A #1	148	<2
*Clanculus margaritarius* A #2	156	Not determined
*Clanculus margaritarius* B #8	53	<6
*Clanculus margaritarius* B #12	<4	<6
*Clanculus pharaonius* #3	73	Not determined

Pyrrole-2,3,5-tricarboxylicacid (PTCA) and 4-amino-3-hydroxyphenylalanine (4-AHP) are specific markers of eumelanin and pheomelanin, respectively. Note *C*. *margaritarius* B specimen #12 was a control for the unpigmented nacreous shell layer, as the pigmented layer was removed by Na-EDTA prior to this analysis.

### Trace elements associated with pigmentation

There was no resolvable pattern of association between trace elements and pigmentation due to either porphyrins or eumelanin. Results for each experiment are given below.

#### Energy Dispersive Spectrometry (EDS) and Microfocus Synchrotron Analysis

We used EDS to determine whether any metals were associated with the shell pigment patterns observed in *C*. *margaritarius*. Fully quantitative analysis was not possible in this case due to extensive beam squirting effect in low vacuum and complex 3D topography, but visual examination of distribution maps for each element showed no strong association of elements with pigments (Figure E in S1 File).

A second attempt was made to search for any correlation between shell pigment patterns and trace metals by using microfocus synchrotron analysis, which has lower detection limits than conventional SEM-EDS. Maps of the trace metals Mn, Fe, Cu, Zn, and Sr show a non-uniform distribution consistent with the SEM-EDS results. The transition metals tend to be distributed in relatively equant granular masses 5 to 150 μm in size. Despite their similarity in distribution patterns there is no obvious correlation between the transition metals and the black, yellow-brown and white regions of the shell (Table F in S1 File). In contrast, Sr does exhibit concentration bands that are parallel to yellow-brown/white colour boundaries (Figure G in S1 File). Point analyses suggest elevated copper concentrations in the black spots on the shell (black spot: 79 ppm, yellow-brown region: 4 ppm, white area: 6 ppm) however the lack of an obvious circular pattern to the copper distribution makes it difficult to conclude that the copper is related to black pigmentation in this case. Furthermore, the yellow-brown coloured region shows a much lower Ca fluorescence than the white or black areas, which suggests that some type of layer of organic matter may overlay the aragonite (the shell-forming mineral, an orthorhombic type of calcium carbonate) and absorb the fluoresced signal. The organic layer is likely periostracum, a thin protein layer found on the outer surface of the shells of most gastropods [55]. Maps of the light elements Si, P, S, and Cl also show granularity at 5 to 150 μm scale, although, in addition, these elements show intensity patterns at the 400 μm scale parallel to the yellow-brown/white banding.

In an attempt to further characterize the shell, sulphur K-edge XANES were collected in the yellow-brown and black regions (Figure H in S1 File). The spectrum from the yellow-brown region exhibits a higher abundance of reduced organic sulphur species than the black area, consistent with the presence of an organic film as postulated to explain the inhibited Ca fluorescence. Absorption of fluoresced X-rays by an organic film might also explain the banding observed for the other light trace elements. The pattern observed for Sr, however, must be due to an oscillatory zonation within the shell itself because the high energy Sr fluorescence would not be appreciably attenuated by an organic layer of the type inferred here.

#### Attenuated Total Reflectance Fourier Transform Infrared Spectra (ATR-FTIR)

ATR-FTIR analyses showed that the black spots and yellow-brown regions include absorption bands that can be assigned to alkanes (2920 and 2850 cm^-1^), amides (1650 cm^-1^) and carboxylate groups (1580 cm^-1^), consistent with the presence of an organic film that includes proteins or protein derived residue, possibly due to the periostracum. The strong CH_3_ (2980 cm^-1^) and C = O (1720 cm^-1^) peaks that dominate a reference uroporphyrin I spectrum are also discernible in the shell (although they are dwarfed by the peaks from the other organic moieties), supporting earlier results (Figure I in S1 File).

#### Time-of-Flight Secondary Ion Mass Spectrometry (ToF-SIMS)

ToF-SIMS also detected hydrocarbons, which appear to correlate with the yellow-brown regions (Figure J in S1 File). The yellow-brown area showed the presence of organic material, aluminium, silicon and calcium (from the shell material). This organic material consists of short hydrocarbon chains, which may be fragments of longer chain material, broken under ion bombardment. A hydrocarbon signal was not produced when the black spots were analysed using this surface sensitive technique.

#### Laser ablation ICP-MS

Laser ablation ICP-MS results showed higher than background levels of strontium and iron for all samples and raised levels of magnesium, copper and barium in some samples but no clear link between colour and metal concentrations (Figure K in S1 File).

## Discussion

### Identification of pink-red and yellow-brown pigments

In this study the dominant shell pigments in *Clanculus margaritarius* and *C*. *pharaonius* were determined to be uroporphyrin I and uroporphyrin III. Both pigments were also found in pigmented foot tissue. The distribution of fluorescence due to uroporphyrin I and III is congruent with visible pink-red pigmentation on the *C*. *pharaonius* shell and at the top of the *C*. *margaritarius* shell, as well as areas of visible yellow-brown pigmentation on later whorls of *C*. *margaritarius*. For ease of discussion here and in future studies we name the pink-red pigment trochopuniceus (‘trocho’ from Trochidae and ‘puniceus’ meaning purplish-red) and the yellow-brown pigment trochoxouthos (‘trocho’ from Trochidae and ‘xouthos’ meaning yellowish-brown).

Uroporphyrin I has been found in the shells (e.g. [16, 23, 36]) and integument of many molluscs (e.g. [56, 57]). On the other hand, uroporphyrin III was not detected in chemical studies of molluscan shells using chromatography on talc and paper [5, 16], probably reflecting limitations of this methodology. It was, however, mentioned in Nicholas and Comfort [23], who cited three studies [35, 58, 59], stating that their findings indicate, “uroporphyrins I and III and traces of coproporphyrin are responsible for the pink fluorescence of molluscan shells” (note that we did not find mention of uroporphyrin III in [58]). Nicholas and Comfort [23], however refuted these findings saying that only one uroporphyrin isomer existed in shells. Later studies do not mention these findings, and argue that only uroporphyrin I occurs in shells (e.g. [5, 37, 60]). Uroporphyrin III has been found only rarely in other groups, most famously in turaco bird feathers [61].

Visible pigmentation as a result of uroporphyrin also occurs in some annelids [62], mammalian teeth [63, 64], the eggshells of some bird species [65, 66] and in the integument of some terrestrial and marine gastropods lacking shells. As a result of these occurrences it has been suggested that the deposition of uroporphyrin is closely connected with calcium metabolism [67], even though it is also known to occur in invertebrate integument and vertebrate skin. It seems likely that only high concentrations of porphyrins result in visible pigmentation and this occurs most frequently in calcified structures. Porphyrin pigments in calcified structures are highly stable over time, with fluorescent patterns detectable in fossil shells as far back as the Jurassic [68].

To date only uroporphyrin is known positively to contribute to shell colour, although trace quantities of other porphyrins have been detected. We identified traces of pentacarboxylate porphyrin in *Clanculus* foot tissue and heptacarboxylate porphyrin in *Clanculus* shells and these porphyrins may correspond to the porphyrins found previously in the shell of bivalve *Pteria radiata* by Fischer & Jordan [69], but not found by later workers [23, 60]. Traces of coproporphyrin were similarly found in both foot tissue and shells of *Clanculus* in our study and have been found in other molluscan shells (e.g. [23, 36, 37]). None of these porphyrins likely contribute to shell colour in *Clanculus*, given the small quantities. Likewise, traces of protoporphyrin have recently been detected using HPLC coupled with mass spectrometry in the shells of three neogastropod species (*Hastula hectica*, *Conus purpurascens*, *Conus ebraeus* and one bivalve, *Argopecten* sp.) [6]. Insufficient quantities were obtained to measure using HPLC and such small amounts suggest that this is unlikely to be the main pigment responsible for shell colour. On the other hand, porphyrins other than uroporphyrin are known to contribute to integumentary colour in some molluscs. For example the red-orange spots on the siphon of the shipworm bivalve, *Bankia setacea* are due to protoporphyrin IX [70]. Protoporphyrin was also found in the foot of an unnamed *Haliotis* (Lockwood, pers. comm. in [60]).

Although *Calliostoma zizyphinum* has shell colours superficially similar to those observed in the *Clanculus* species, the pigmentation is not due to uroporphyrin. Only traces of uroporphyrin I and III were identified in HPLC analyses of *Ca*. *zizyphinum* shell and no fluorescence was observed under UV light. Another difference is that the colour in *Ca*. *zizyphinum* shells fades over a period of months, whereas the shell colour in the two *Clanculus* species is stable over the same time period. Based on published studies of trochoidean shells, it is possible that the pink-red and yellow-brown colours in *Ca*. *zizyphinum* are due to bilins as these pigments have been found in other species from the same superfamily (e.g. trochids, turbinids and haliotids; [25, 36]). Further biochemical investigation is required to confirm this hypothesis.

### Same porphyrins–different colours

Red fluorescence under UV light is typical of porphyrins and occurs only rarely in other (mostly unidentified) molluscan pigments ([15] and references therein). Molluscan species where red fluorescence has been shown to broadly coincide with visible pigment patterns on the shell include several different visible colours. In such cases, the visible pigment may be black (*Cittarium pica*), scarlet or pink-red (*Tectus niloticus*, *Clanculus puniceus*, *C*. *pharaonius*), pink-red or yellow-brown (*C*. *margaritarius*), pink (*Aplustrum amplustre*), brown (*Pteria*) [15] (this study). Only in the case of *C*. *margaritarius* and *C*. *pharaonius* has the visible pigment been shown to be congruent with the distribution of uroporphyrin I and uroporphyrin III.

Porphyrins can associate with different metal ions that may affect the colour of the pigment; however, in this study we found no clear evidence that the pattern of distribution of metal ions matched shell colour. Moreover, the UV-visible spectra of shell extracts suggest that metal-free porphyrins are dominating, although extraction methods using acid can affect metal content. This is not likely to be an issue in our case, as protonation with acid of central nitrogens in the macrocycle causes the same change in UV-visible spectra as metalation; essentially, the four Q-bands collapse to two [50], which is not what we found. Moreover, porphyrins extracted with EDTA at near-neutral pH (as in one of our specimens) are more likely to preserve the original molecular configuration than those extracted with acid [6].

A more compelling hypothesis for the difference in colour between trochopuniceus and trochoxouthos is suggested by studies of the turaco bird. This brightly coloured bird has green and red feathers. The pigment responsible for the red colour is known as turacin and that for the green pigment is turacoverdin. Turacin was identified as uroporphyrin III complexed with copper [61, 71, 72] and several authors suggest that turacoverdin is ‘identical to or closely related to an oxidised form of turacin’ [73]. Our tests showed that while the colour of trochoxouthos extracted by Na-EDTA was stable over 13 d, an extract of trochopuniceus was initially scarlet-red but turned red-brown over time becoming more similar in colour to trochoxouthos, consistent with the spectral changes observed during a 13 day aging in the presence of light (initially) and oxygen. An explanation for this change is suggested by the difference in Q-bands observed in the spectra for the two pigments and the two different coloured solutions of trochopuniceus. In the case of an unmetallated porphyrin, the Q-bands give some indication of the substituents on the pyrrole β- and meso-positions. Changing Q-bands suggest changes to these substituents, which is known to affect colour [50].

### Black pigment

We hypothesised that black spots on *Clanculus* shells are the result of melanin. Melanins are considered to be the most perplexing of all pigments [74, 75]. They cannot be described in terms of a single, well-defined structure and fundamental gaps in our understanding have resulted from a lack of standardised procedures for their extraction, purification, physical, spectral and chemical characterization and use in experimental studies [74, 75]. They are broadly defined as “pigments of diverse structure and origin derived by the oxidation and polymerization of tyrosine in animals or phenolic compounds in lower organisms” [75]. Two types of melanin, likely derived from tyrosine, are known to occur in molluscs. The first, eumelanins, occur widely in vertebrates and invertebrates and are responsible for brown and black colours [76]. Pheomelanins are macromolecules that contribute to the colour of human red hair, and although most commonly known in mammals and birds they have recently been identified as the screening pigment in the shell-eyes of chitons [77]. Pheomelanins may be brown, yellow or red in colour.

Our HPLC studies identified small amounts of eumelanin in *Clanculus* shells, but no tests were undertaken to identify where this pigment is located. Based on colour, the eumelanin likely occurs in areas with black spots. This hypothesis is further supported by initial examination of the *Clanculus* shells under a UV light, which revealed no fluorescence for areas with black pigmentation and in nearby white areas. Non-fluorescent white areas presumably lack fluorescent (and visible) pigment. On the other hand, melanins are known to produce dark pigmentation that quenches fluorescence [13].

It is known that eumelanin has a large capacity for binding metal ions and recent studies have shown a correlation between the distribution of copper and eumelanin in several extant and extinct species (e.g. [78]), although this may not apply in all cases (e.g. [79]). EDS point analyses in this study identified elevated copper concentrations in the black spots on the shell of *C*. *margaritarius* when compared with yellow-brown or white areas, but the lack of an obvious circular pattern to the copper distribution makes it difficult to conclude that the copper distribution is controlled by black pigmentation alone.

### Comparison of methods for the identification of shell pigments

Although results of HPLC analyses, UV-visible spectroscopy and fluorescence studies provide positive evidence of porphyrin pigmentation, Raman spectra of *Clanculus* shells do not provide a definitive identification of shell pigments. Raman spectra alone cannot resolve specific molecular structure [7], but conjugated polyenes, such as polyenals and carotenoids, have been described from molluscan shells by the analysis of Raman bands. Such studies are becoming increasingly popular for the identification of shell pigments (e.g. [7, 27–30, 51, 54, 80, 81]) and we identify here some potential issues with distinguishing between polyenes and porphyrins.

Raman bands at ~1555 cm^-1^ such as those observed in the *in situ* spectra of the *C*. *margaritarius* shell can be attributed to porphyrins due to a C_β_-C_β_ stretching mode in pyrrole systems [29, 52, 53], but have also been assigned to conjugated polyenes. The observation of Raman bands at ca. 1500–1530 and 1150–1160 cm^-1^ is due to stretching vibrations of the single and double carbon bonds, as sharp and well defined bands, showing strong dependence with the laser excitation; such bands can be easily assigned to conjugated polyenes, such as carotenoids or similar compounds, whereas broad and low defined bands are suggestive of other types of pigments containing conjugated bonds, such as porphyrins. Based on our previous experience, it seems likely that if porphyrins and carotenoids co-occur, only the carotenoids would be detected using Raman spectroscopy

Conjugated polyenes, such as polyenals and carotenoids produce characteristic vibrational bands at *ca*. 1500–1570, 1120–1160 and 1000–1020 cm^-1^, and have been attributed to n(C = C), n(C−C) and r(C-CH_3_) or n_4_(C-CH_3_) respectively [7, 27–29]. The main differences between polyenals and carotenoids are the red-shifted wavenumber position of the (C−C) stretching mode of polyenals by *ca* 30 cm^−1^ when compared with that of carotenoids, as well as the absence in polyenals of the deformation mode related to the r(C−CH_3_) group at *ca* 1000 cm^−1^ and the presence of n_4_(C−CH_3_) at *ca* 1020 cm^−1^ [82, 83].

Carotenoid-like Raman bands were reported for shell pigments in the trochid ‘*Gibbula sp*’, although the authors’ state that they differ from the “partially or completely demethylated polyenes” also referred to as conjugated polyenals found in their study in a variety of caenogastropods, two scallops and one venerid bivalve [28]. We would not predict that carotenoids would occur in trochids based on the systematic relationship with pigments observed to date [4], but our study confirms that it may be difficult to use Raman spectroscopy to distinguish between conjugated polyenes and porphyrins (although see [84]).

It is important to point out that conjugated polyenes extracted from biological samples or synthetic products (parrodienes) are often only weakly soluble in organic solvents [83, 85], which limit the use of many techniques for pigment identification. This, in combination with difficulties associated with isolating enough pigment for chemical analyses, explains why Raman spectroscopy has proved a valuable tool for the identification of chemical compounds directly from tissues or surfaces of diverse materials.

Surface-sensitive methods used in this study were affected by organic layers occurring on top of the shell and as such may not be the most suitable method for identifying shell pigments. The organic material detected in this study on the surface of the shell likely corresponds to the periostracum, a thin protein layer found on the outer surface of the shells of most gastropods [55]. A protein coating has also prevented successful ATR-FTIR identification of underlying materials in other studies (e.g. [86]). Problems with surface contamination and overlying organic layers might be avoided in future studies by using a cross section of shell.

### Evolution of pigments in Mollusca

Several authors have suggested that shell colour in gastropods, and the assimilation of porphyrins in particular, arose originally through the need to eliminate ‘unmanageable’ metabolic waste products [13, 87], although others have contested this idea [7]. The idea that shell pigments were originally waste products provides a potential starting point for the stepwise evolution of shell colour and pattern in molluscs. The long-term evolutionary maintenance of porphyrin pigmentation in combination with the likely energetic cost involved in porphyrin production [4], suggests these pigments offer some selective advantage. Since the Cambrian explosion and the development of the first image-forming eyes, there has likely been strong pressure on shell colour and pattern to serve as camouflage or as warning as predators evolved both colour vision and better visual acuity [87]. However, visible colours associated with higher concentrations of porphyrins and their pattern of deposition likely came under strong selection in the evolutionary past even before image-forming eyes had evolved. For example, it has been suggested that porphyrins may offer thermal advantages to bird eggs in warm, exposed environments as they do not absorb infrared radiation [39]. Intertidal marine snails exposed to high temperatures, with porphyrin pigmentation in their shells might also be expected to benefit from this property, although this has not been tested.

Although there have been no studies of the evolution of shell colour in Mollusca, in part given the inherent difficulties of fully characterising shell pigments, data available to date hint at the possibility they may be distributed in a phylogenetically relevant pattern [4]. Yet, despite the fact that Trochidae and Calliostomatidae are sister taxa [88], three species with superficially similar colouration from these two families do not share the same pigments. This has important ramifications for studies of the molecular processes involved in the synthesis of shell colour, as genes involved in colour production may not be shared among taxa.

It also suggests that colour may be a poor taxonomic character for comparisons among genera and families because similar colours can evolve by convergence. The fact that different pigments produce similar colours in the three different species examined in this study suggests that there may be some adaptive value for these colours and patterns despite differences in their habitats. Red fluorescence produced by porphyrins, on the other hand, is unlikely to be visually selective in brightly lit conditions, since it is only apparent in very strong UV light, which the animals tend to avoid (living in underwater caves or under rocks).

### Conclusions

This is the most rigorous investigation into the identification of molluscan shell pigments undertaken to date. As a result of these studies the dominant shell and foot pigments in *C*. *margaritarius* and *C*. *pharaonius* are shown to be pigments that we name trochopuniceus and trochoxouthos. These pigments correspond to the visible colours pink-red and yellow-brown respectively. They are both comprised of uroporphyrin I and III. Their difference in colour is probably consistent with differences in the relative intensity of the Q-bands in the UV-visible spectra of porphyrins, but further research is needed in this direction. Traces of eumelanin were also identified and likely correspond to small black spots on the shell. The absence of trochopuniceus and trochoxouthos from *Ca*. *zizyphinum*, despite similar colours on its shell, confirms that similar shell colours can arise from different pigments [7]. This important fact highlights the need to identify pigments chemically prior to evolutionary studies, as mistakes might be made regarding homoplasy *versus* homology if one looks only at colour and pattern. Our results also have important implications for future studies of shell pigments. Our study shows that HPLC, fluorescence studies and UV-visible spectrometry were the most informative methods for identifying eumelanin and uroporphyrin I and III.

## Supporting Information

S1 File Compressed zip folder containing two tables and nine figures. **Table A. Table with sample details for *Clanculus margaritarius* A & B, *C*. *pharaonius* and *Calliostoma zizyphinum*.** Specimen number, sampling locality, analyses undertaken in this study, and corresponding photos or data. **Fig B. Confocal images showing uroporphyrin distribution in areas of pink-red pigmentation for *Clanculus pharaonius*.** Shell images on each line are of a single shell and are in approximately the same orientation and magnification. (A-C) Top of shell (specimen #4). (A) Visible pink-red pigmentation. (B) Shaded volume 3D projection of a spectrally mixed image of the same shell showing fluorescent areas congruent with visible pink-red pigment. (C) Maximum intensity projection of a spectrally unmixed image showing distribution of uroporphyrin I and III. (D-F) Area on later whorl of same specimen. (D) Visible pigmentation for approximate areas used in analyses. (E) Maximum intensity projection of spectrally mixed images showing fluorescent areas under UV light congruent with pink-red pigmentation. (F) Maximum intensity projection of spectrally unmixed images showing distribution of uroporphyrin I and III. Note that in unmixed images green corresponds to uroporphyrin I and red to uroporphyrin III and yellow to areas where both co-occur. Note also that fluorescence is quenched in black spots and nearby white areas. Visible pigmentation is marked on each image: pr–pink-red, bl–black spot, w–white area. **Fig C. Results of Raman spectroscopy.** Spectra excited by 532 nm light on surface of shell of *Clanculus margaritarius* B (specimen #7). (A) Yellow-brown area. (B) Black spot. (C) Comparison of two spectra from (A) and (B). **Fig D. High performance liquid chromatography (HPLC) analyses for eumelanin in *Clanculus margaritarius* B.** (A) Chromatogram of specimen #8 (measurable PTCA). (B) Chromatogram of specimen #12 (no obvious PTCA). **Fig E**. **EDS maps of *Clanculus margaritarius* shell (specimen #11) for 14 metal ions.** Black spot spectra were taken from the centre of the ESEM images; yellow-brown spectra were taken from the areas in the top left corner. Analyses were run on a FEI Quanta 650 FEG SEM equipped with a multi-channel Bruker 5060F XFlash QUAD EDS system with an acquisition time of 180 s, working distance 8.8 mm, chamber pressure 67 Pa and an accelerating voltage of 25 kV. **Table F. Synchrotron XRF EDS quantification of pigmented and unpigmented regions of *Clanculus margaritarius* (specimen #11).** 2σ errors in parentheses. Values in ppm unless where stated in weight% (%). **Fig G. Microfocus map of strontium (Sr) in *C*. *margaritarius* shell (specimen #11).** Sr exhibits concentration bands that are parallel to yellow-brown/white colour boundaries. Optical photo not available. **Fig H. Sulfur XANES spectra obtained from the yellow-brown and black regions of a *C*. *margaritarius* shell (specimen #11).** The spectrum from the yellow-brown region exhibits a higher abundance of reduced organic sulfur species than the black area, consistent with the presence of an organic film as postulated to explain the inhibited Ca fluorescence. Vertical dashed line represents the peak position for sulfate determined by a ZnSO_4_ standard (~2482 eV). **Fig I. Attenuated Total Reflectance Infrared Spectra of *C*. *margaritarius* (specimen #11) bulk shell material compared to a dark spot and a uroporphyrin I standard.** Dotted lines indicate potential peaks of similarity between the samples though this is not enough to demonstrate the presence of uroporphyrin, there are also no apparent differences between the spectra of the bulk shell material and the dark spot. Both show the presence of organic matter and protein moieties. **Fig J. TofSIMS maps of *Clanculus margaritarius* shell (specimen #11).** Secondary ion images are shown with a rainbow colour scheme from black (no counts) to red (maximum). Numbers in parentheses are the total counts given in each individual image followed by the maximum count. In positive secondary ion mode, non-polar organic molecules usually ionize as (M+H)+ which means one hydrogen atom has to be subtracted from the measured secondary ions to get the actual molecule present in the sample. **Fig K. Graphs showing results of laser ablation for five selected metals**. Each curve represents a different sample. There are seven samples for black pigment (black lines), three from *Clanculus margaritarius* and four from *C*. *pharaonius* shells; five samples for red pigment (red lines) from *C*. *pharaonius* shells; 12 samples for white areas (grey lines), seven from *C*. *margaritarius* and five from *C*. *pharaonius* shells; and nine samples for yellow-brown pigment (yellow lines) all from *C*. *margaritarius*. See Table A for list of specimens used.
(ZIP)
