# Supplementary material for: Identification of Shell Colour Pigments in Marine Snails Clanculus pharaonius and C. margaritarius (Trochoidea; Gastropoda)
Source: PLoS One. 2016 Jul 1;11(7):e0156664. doi: 10.1371/journal.pone.0156664 (PMC4930200; doi:10.1371/journal.pone.0156664)
Supplement: S1 File — Table A. Table with sample details for Clanculus margaritarius A & B, C. pharaonius and Calliostoma zizyphinum. Specimen number, sampling locality, analyses undertaken in this study, and corresponding photos or data. Fig B. Confocal images showing uroporphyrin distribution in areas of pink-red pigmentation for Clanculus pharaonius. Shell images on each line are of a single shell and are in approximately the same orientation and magnification. (A-C) Top of shell (specimen #4). (A) Visible pink-red pigmentation. (B) Shaded volume 3D projection of a spectrally mixed image of the same shell showing fluorescent areas congruent with visible pink-red pigment. (C) Maximum intensity projection of a spectrally unmixed image showing distribution of uroporphyrin I and III. (D-F) Area on later whorl of same specimen. (D) Visible pigmentation for approximate areas used in analyses. (E) Maximum intensity projection of spectrally mixed images showing fluorescent areas under UV light congruent with pink-red pigmentation. (F) Maximum intensity projection of spectrally unmixed images showing distribution of uroporphyrin I and III. Note that in unmixed images green corresponds to uroporphyrin I and red to uroporphyrin III and yellow to areas where both co-occur. Note also that fluorescence is quenched in black spots and nearby white areas. Visible pigmentation is marked on each image: pr–pink-red, bl–black spot, w–white area. Fig C. Results of Raman spectroscopy. Spectra excited by 532 nm light on surface of shell of Clanculus margaritarius B (specimen #7). (A) Yellow-brown area. (B) Black spot. (C) Comparison of two spectra from (A) and (B). Fig D. High performance liquid chromatography (HPLC) analyses for eumelanin in Clanculus margaritarius B. (A) Chromatogram of specimen #8 (measurable PTCA). (B) Chromatogram of specimen #12 (no obvious PTCA). Fig E. EDS maps of Clanculus margaritarius shell (specimen #11) for 14 metal ions. Black spot spectra were taken from the centre of the ESEM [file pone.0156664.s001.zip › S1File/Table F in S1 File.docx]

**Table F. Synchrotron XRF EDS quantification of pigmented and unpigmented regions of *Clanculus margaritarius* (specimen #11).**

2σ errors in parentheses. Values in ppm unless where stated in weight% (%).

|  | **Black** | | **Yellow-brown** | | **White** | |
| --- | --- | --- | --- | --- | --- | --- |
| **Ca** | 48.9% | (3.56%) | 6.6% | (0.86%) | 46.8% | (3.8%) |
| **Ti** | 63 | (13) | 32 | (7) | 89 | (18) |
| **V** | 20 | (4) | 11 | (2) | 12 | (3) |
| **Mn** | 15 | (3) | 4 | (1) | 10 | (2) |
| **Fe** | 117 | (17) | 39 | (7) | 179 | (24) |
| **Ni** | 4 | (1) | 3 | (1) | 4 | (1) |
| **Cu** | 79 | (8) | 4 | (1) | 6 | (1) |
| **Zn** | 4 | (1) | 1 | (1) | 4 | (1) |
| **Sr** | 1030 | (21) | 817 | (18) | 965 | (23) |
